# Magnetic core-modified silver nanoparticles for ibuprofen removal: an emerging pollutant in waters

**DOI:** 10.1038/s41598-020-75223-1

**Published:** 2020-10-26

**Authors:** Yesica Vicente-Martínez, Manuel Caravaca, Antonio Soto-Meca, Rubén Solana-González

**Affiliations:** University Centre of Defence At the Spanish Air Force Academy, MDE-UPCT, C/Coronel López Peña S/N, Santiago de La Ribera, 30720 Murcia, Spain

**Keywords:** Environmental chemistry, Chemical engineering

## Abstract

In this work we present a novel procedure for ibuprofen adsorption from waters employing magnetic core-modified silver nanoparticles. We demonstrate that 93% adsorption of ibuprofen is achieved in 45 min by means of a simple method, for neutral pH and room temperature, also using a low dose of adsorbent, equal to 7 mg in 500 µL of suspension. The characterization of the adsorbent, before and after adsorption, was carried out by means of field emission scanning electron microscopy, energy dispersive X-ray spectroscopy, BET analysis, Fourier-transform infrared spectroscopy and differential scanning calorimetry. It is worth pointing out that ibuprofen can be desorbed and the adsorbent can be reused, remaining unaltered for the first three cycles, and showing 89.3% adsorption efficiency after the third regeneration. A three-parameter model and the Langmuir isotherm characterize the kinetics and isotherm of adsorption.

## Introduction

Pharmaceuticals are products used in large doses in daily life considered as contaminants of emerging concern. Due to the large amounts of drugs consumed, the hydrogenic sources suffer from contamination processes that give rise to toxicological effects in humans despite its low concentrations^1,2^. Many medicines considered as emerging contaminants are constantly detected in groundwater, wastewater treatment plants and water supply. The inefficiency of conventional methods used in water treatment plants to remove the contaminant motivates the development of effective methods to treat effluent contamination^3^.

According to the physico-chemical properties of drugs, their degradation products and the characteristics of the soils, these substances can reach the groundwater and contaminate the aquifers or remain retained in the soil, thus affecting the ecosystem and humans through the food chain^4^. Additionally, the portion of medicines not assimilated by the organism, as well as chemical substances administered to animals, usually become part of wastewater. Consequently, different ways of removing medicines in waters have been studied^5^.

Recently, Ibuprofen (IB), a medicine from the family of non-steroidal anti-inflammatories, used against muscle problems and inflammatory disorders, has become an important research focus, since it is an emerging micro-pollutant with a high economic and environmental impact^6^. It is widely used in society, presenting a chemical structure not easily degradable, being eliminated from the body through urine. Due to this, it can be found in water samples of different origins, thus even affecting the swimming capacity of aquatic species and, therefore, its ability to move, feed and reproduce^7,8^.

Some of the methods to eliminate IB from water samples are based on its degradation or oxidation. In the former case, it is worth pointing out the employment of microorganisms through biodegradation^9^, biotransformation^10^, photocatalytic process with TiO_2_^11^ and coagulants methods^12^. In the latter case, IB is oxidized by using sulfates thermally assisted by ultraviolet light^13^. Other relevant methods include the use of peroxymonosulfate activation under visible-light irradiation^14^, graphene oxide based heterogenous catalytic ozonation^15^, strong nano-clay composite^16^ or electro-fenton process^17^.

Notwithstanding, the methods based on adsorption have been the most used in recent years to remove IB from aqueous media due to its high removal efficiency, being the adsorbents of very diverse nature. Ibuprofen has been adsorbed on Organo-Zeolite^18^, activated carbon cloths^19^, mesoporous silicon microparticles^20^, activated carbon^21–23^ or mica and montmorillonite^24^, polymeric resins^25^, functionalized materials^26^, waste-based adsorbents^27^, or biosorbents^28^, among others. Some current methods employed to remove ibuprofen from aqueous samples are very laborious and require long times to reach the results^29,30^, other procedures demand large amounts of adsorbent^31^ or high temperatures. However, in the present method high adsorption efficiency is achieved under mild experimental conditions such as neutral pH and room temperature. Furthermore, it takes only a few minutes to achieve the complete removal of ibuprofen.

In particular, nanoparticles have been employed in recent studies to remove different pollutants^32,33^ from water. Particularly, these have been employed to remove IB from different media due to its very small size and high contact surface, thus achieving high adsorption efficiency^34–36^. In this work, magnetic core-modified silver nanoparticles (Fe_3_O_4_@AgNPs) have been used to adsorb ibuprofen from aqueous solutions, being a new outstanding method for reaching high removal efficiency, under very mild conditions and in a short time compared with other procedures^21,37,38^. Fe_3_O_4_@AgNPs are easily synthetized and removed from media using a magnet. Additionally, IB can be desorbed from the nanoparticles for them to be reused.

## Experimental

### Materials and instrumentation

Pure water obtained with a Millipore system (Millipore, Bedford, MA, USA) was used exclusively. All the solutions were stored in polypropylene or polytetrafluoroethylene vessels. Analytical grade acetonitrile was obtained from Panreac. The standard IB and the other chemicals used were purchased from Sigma-Aldrich.

The concentrations of IB were determined by HPLC (high performance liquid chromatography) on a JASCO BS-4000 system equipped with a sample injector and an ultraviolet detector at 254 nm. A C18 column (150 × 4.6 mm i.d., 5 µm) was used. It was employed as a mobile phase 80% acetonitrile and 20% phosphoric acid solution at pH 3. The flow rate was 1 mL min^−1^ and the injection volume was 10 µL. The reproducibility and repeatability of the HPLC method were checked by means of an IB standard solution.

Permanent Nd–Fe–B magnets were supplied by Supermagnete (Gottmadingen, Germany). The agitator used to manufacture the nanoparticles was a polyethylene shovel agitator purchased from Argolab (AM20-D model).

### Preparation of magnetic core-modified silver nanoparticles (Fe_3_O_4_@AgNPs)

20 mL of water was heated at 80 °C and continuously stirred under nitrogen atmosphere. Then, 0.56 g FeCl_3_∙6H_2_O and 0.2 g FeCl_2_∙4H_2_O were added. When the solids were dissolved, 2 mL of concentrated ammonia solution were incorporated and the solution was stirred for 10 min. The particles were separated using a permanent magnet and the supernatant was discarded. The solid was washed three times with water until the washing liquids were neutral. The iron oxide nanoparticles obtained in this way (approximately 0.28 g Fe_3_O_4_) were suspended in 20 mL of water. Then, 5.7 mL of diluted silver nitrate solution (0.011 g L^−1^) were added, the mixture was stirred for 5 min and using the magnet were separated and washed several time with water. Finally, these particles (Fe_3_O_4_@AgNPs) were suspended in 20 ml of water again^39^.

**Figure 1:**
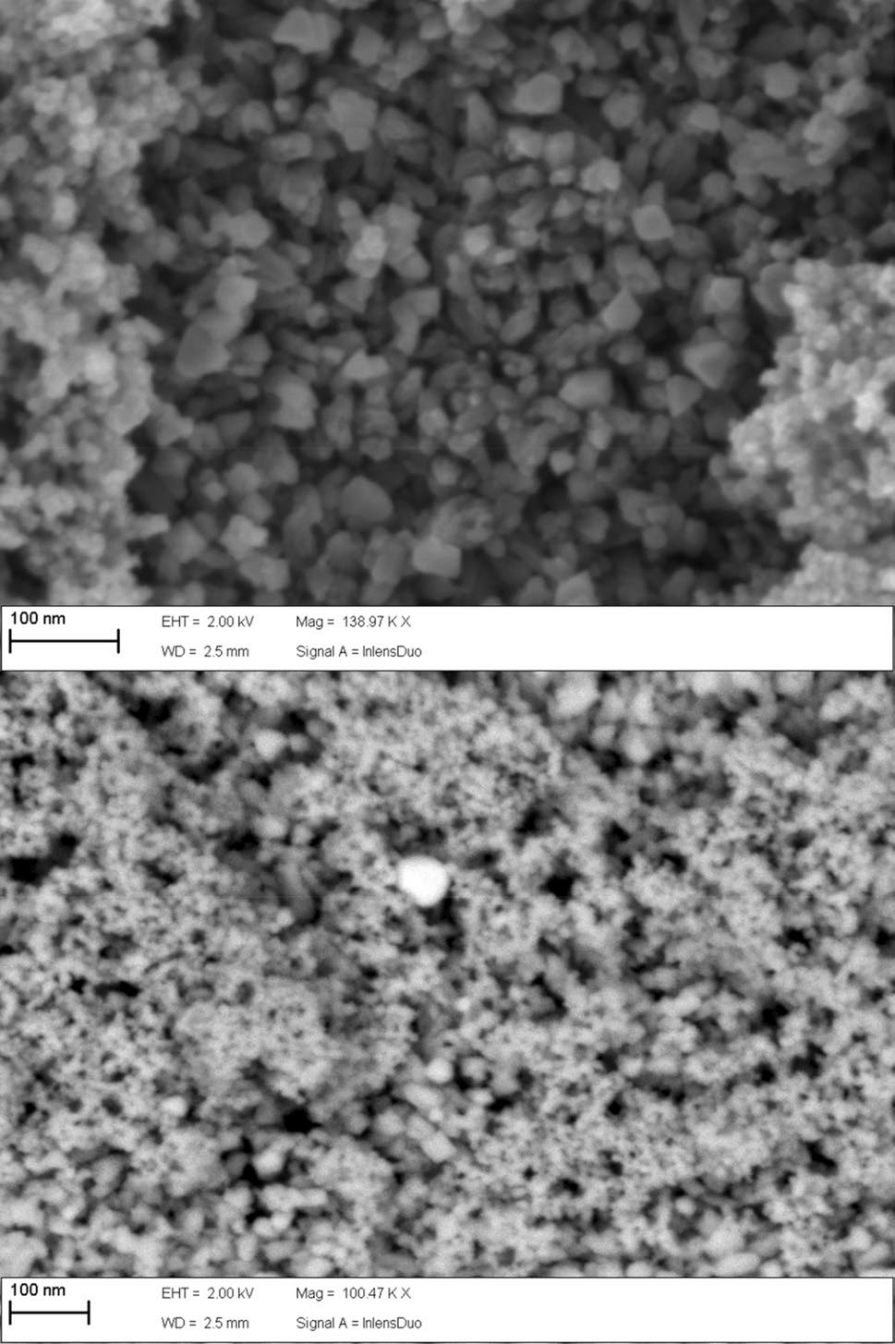
FESEM images for Fe_3_O_4_ (3D, top) and Fe_3_O_4_@AgNPs (intensity, bottom). The latter reveals the presence of Ag in the sample.

**Figure 2:**
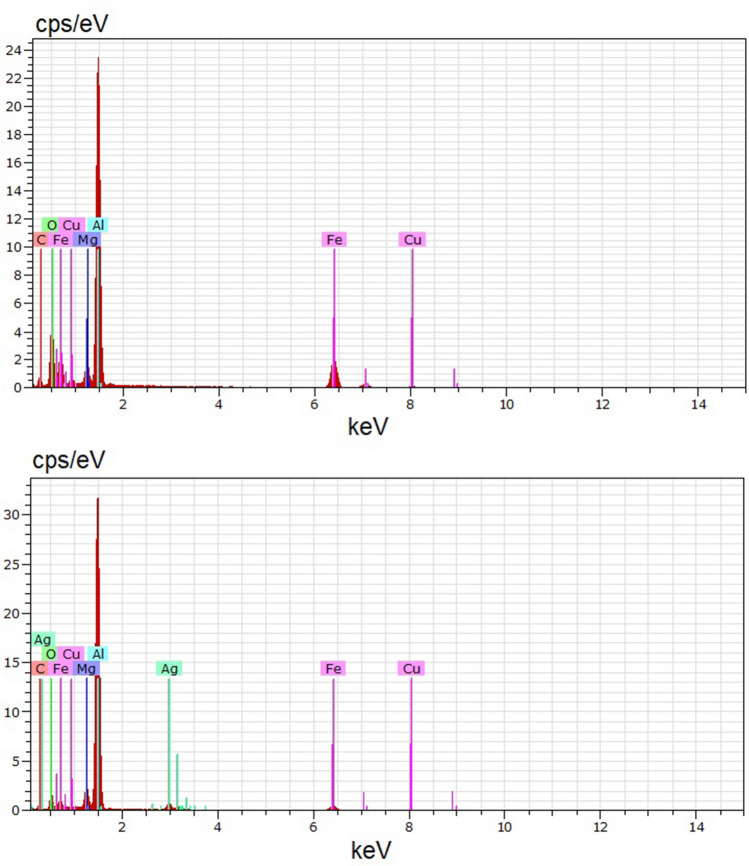
EDX graphs for Fe_3_O_4_ (top) and Fe_3_O_4_@AgNPs (bottom) obtained from the FESEM analysis of Fig. 1. In the latter, peaks corresponding to Ag appear in the plot, checking the presence of Ag in the sample.

A Field emission scanning electron microscopy analysis (FESEM) was performed to check the presence of Ag in the samples. In Fig. 1 (top) it is shown the 3D image for Fe_3_O_4_, while Fig. 1 (bottom) shows the intensity image for Fe_3_O_4_@AgNPs, where the Ag appears as spherical, and shiny due to its high atomic number. Additionally, energy dispersive X-ray spectroscopy (EDX) graphs for Fe_3_O_4_ and Fe_3_O_4_@AgNPs are presented in Fig. 2, top and bottom, respectively. In the latter, the signal corresponding to Ag appears in the plot.

A BET test was carried out to determine the contact surface area of the adsorbent, giving a value equal to 116.476 m^2^/g, and a correlation coefficient for the BET isotherm of 0.999.

## Results and discussion

### Ibuprofen adsorption procedure.

A water sample (10 mL) containing IB at a concentration of 0.2 mg L^−1^ was placed in a polypropylene tube, 500 µL of Fe_3_O_4_@AgNPs suspension were added. After shaking 30 min at *T* = 298 K, the magnet was placed at the bottom of the tube for 5 min and the adsorbent was separated. The supernatant was analyzed by high performance liquid chromatography to determine the maximum removal efficiency of IB.

Several studies have been performed to check the presence of IB in Fe_3_O_4_@AgNPs samples after adsorption. Fig. S1 (see Supplementary Material) shows a Fourier-transform infrared spectroscopy (FTIR) spectrum for Fe_3_O_4_@AgNPs after the adsorption process, where the characteristic IB signals are marked (carbonyl group at 1704,12 cm^−1^; stretch frequencies of Csp^3^-H of isobutyl group at 2951,93 and 2922,15 cm^−1^; aromatic C = C bond at 1560,83 cm^−1^; O–H bond at 3100 cm^- 1^). In Fig. S2 (top) (see Supplementary Material), a differential scanning calorimetry (DSC) analysis for pure IB is shown, while in Fig. S2 (bottom) (see Supplementary Material) the same study is presented for IB adsorbed onto Fe_3_O_4_@AgNPs. In the latter, the double peak presented in the striped area reveals the strong interaction between the species.

Regarding the effect of temperature on the adsorption process, it was found that for *T* = 298 and 303 K, the adsorption efficiency reaches the same value, maximum. For *T* greater than 303 K, Fe_3_O_4_@AgNPs is dissolved in the media. The choice for the adsorption procedure was then 298 K. However, in “Adsorption isotherms” we perform a thermodynamic study for temperatures above 303 K, once the Fe_3_O_4_@AgNPs is dissolved, in order to complete the characterization adsorption process of the involved species.

### Effect of pH on IB adsorption

**Figure 3:**
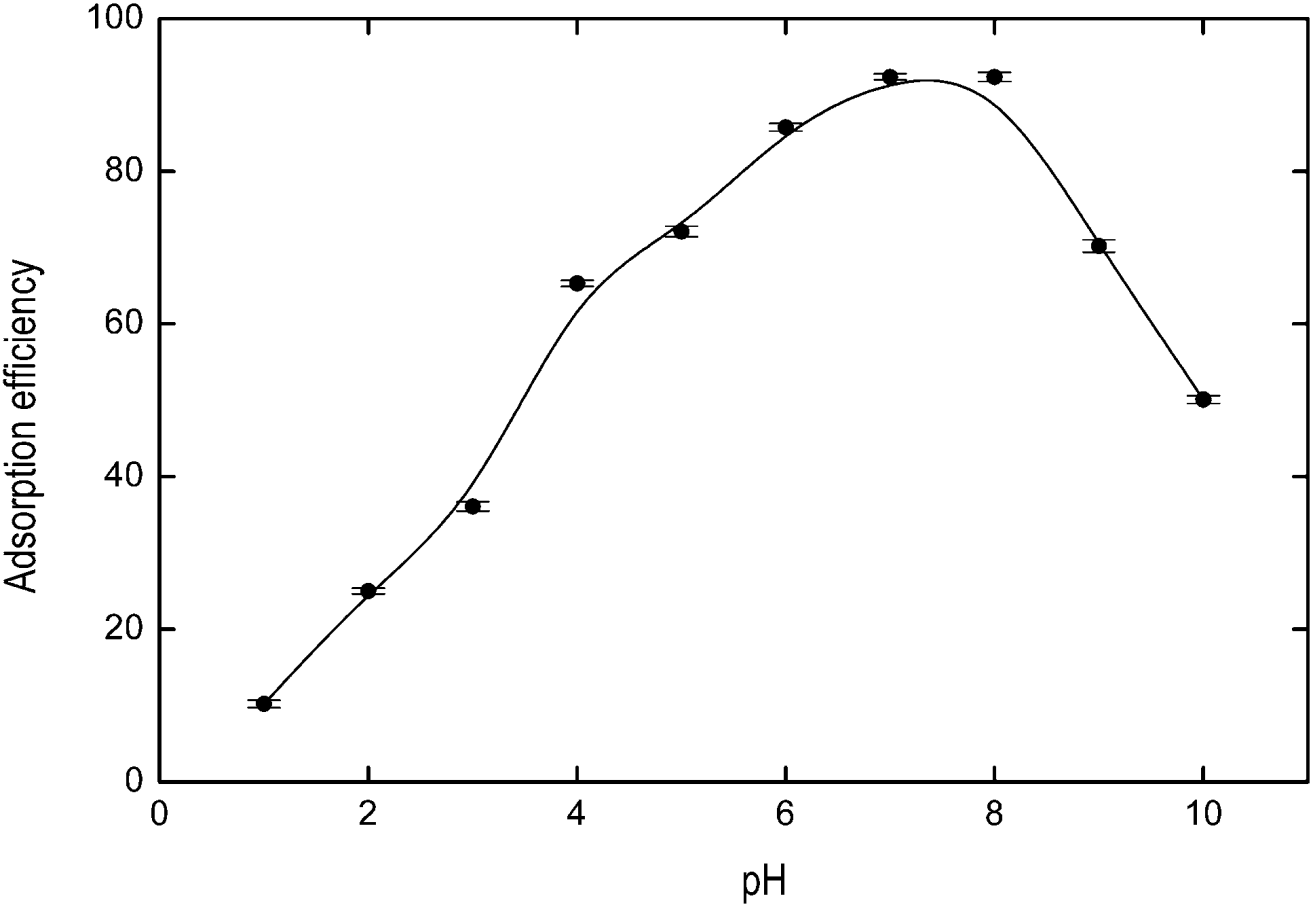
Effect of pH on the ibuprofen adsorption.

The effect of pH on the adsorption of IB on magnetic core-modified silver nanoparticles was studied within the range 1–10. Figure 3 shows that the highest degree of adsorption was achieved at pH 7. The dependence of adsorption on pH is associated with the point of zero charge (PZC) of the adsorbent (Fe_3_O_4_@AgNPs) and the pKa of the IB. The PZC is 6.93 for Ag^40^. The IB is a weak acid (pKa = 5.2) and exists as a neutral species por pH < pKa, coexists as neutral and anionic species at pH ≅ pKa, and exists as anion species for pH > pKa^41^. For pH > 8 IB is deprotonated and Ag surface becomes negatively charged, thus leading to an electrostatic repulsion which reduces the adsorption efficiency^37^. For pH values greater than pKa but lower than PZC, electrostatic attraction between anionic ibuprofen and the positively charged surface of silver nanoparticle improves the adsorption capacity^42^. For pH < pKa IB is mainly in its protonated form (non-ionized) and Ag surface is positively charged, being adsorption mainly conducted by Van der Waals or hydrogen bonding interactions^43^.

An increase in the ionic strength of the solution reduces the electrostatic interactions, either attractive or repulsive, due to a screening effect of the surface charge produced by the reduction of pH^44^. This presumably explains the reason why increased ionic strength resulted in the decreased sorption of ibuprofen in the low pH range^45^. The ionic strength could affect the activity coefficients hindering the transfer from the solution to the adsorptive surface^46^.

### Effect of adsorbent volume

**Figure 4:**
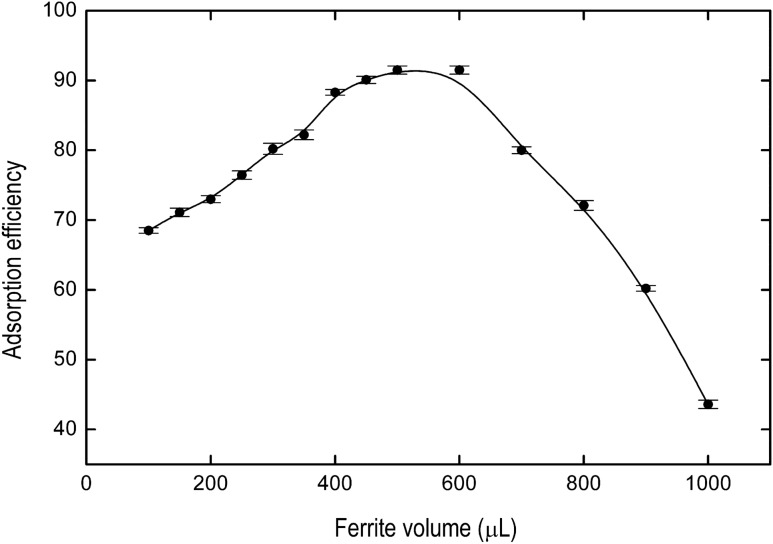
Dependence of the adsorption efficiency on Fe_3_O_4_@AgNPs suspension volume for an ibuprofen concentration equal to 0.02 mg L^−1^. Solid line represents B-spline connectors.

The volume of Fe_3_O_4_@AgNPs suspension has been studied from 100 to 1000 µL for IB concentration of 0.02 mg L^−1^. The results are shown in Fig. 4. As it is depicted, the volume necessary of Fe_3_O_4_@AgNPs to reach the maximum adsorption efficiency is 500 µL. From 900 µL, the percentage of adsorption decreases due to the high volume of adsorbent in the medium and it is not completely removed with the magnet.

### Contact time effect

**Figure 5:**
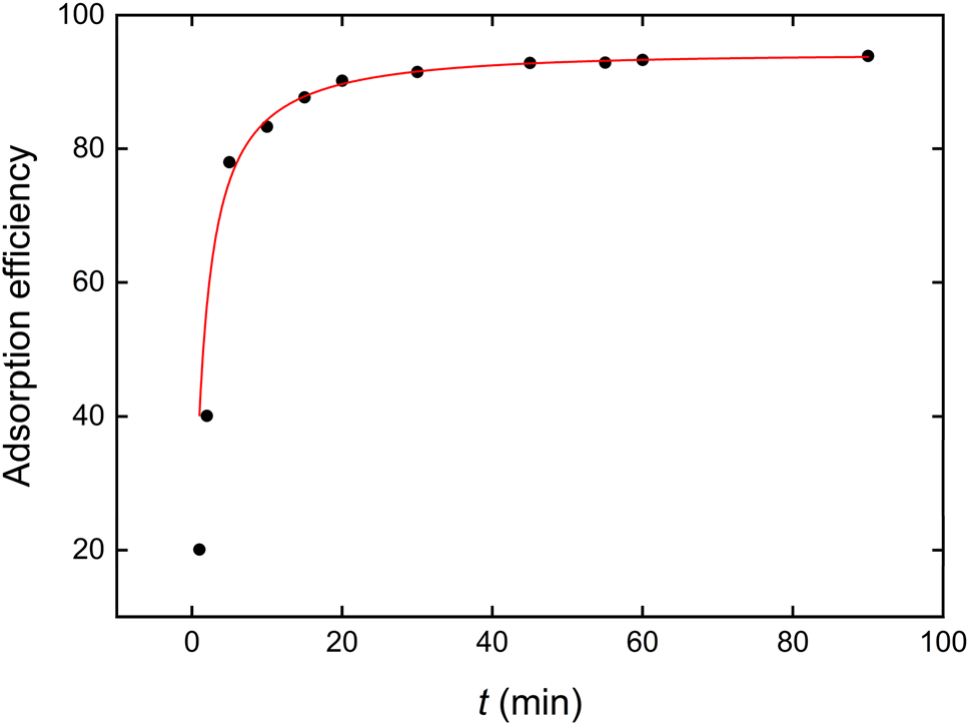
Effect of incubation time on the IB adsorption efficiency. Solid line represents the fit of experimental data to Eq. (1a).

The contact time between Fe_3_O_4_@AgNPs and the solution containing IB was studied from 1 to 90 min in order to achieve the maximum adsorption efficiency, reached at 45 min. From there, the percentage of adsorption remains constant until 90 min, as shown in Fig. 5. The solid line represents the fit to the efficient hybrid combined first and second order three-parameter model^47^:

1a $$Adsorption\,\, efficiency \left( {AE} \right) = \left( {\alpha - \beta } \right)\frac{{\left( {\beta /\alpha } \right)e^{{\left( {\beta - \alpha } \right)\gamma t}} }}{{\left( {\beta /\alpha } \right)e^{{\left( {\beta - \alpha } \right)\gamma t}} - 1}} + \beta$$

where $\alpha$, $\beta$, $\gamma$ are characteristic paremeters. The fit was carried out via software Origin 2019, offering $R^{2} = 0.99986$ and recuded $\chi^{2} = 0.39$. Although this model is credited to be consistent with equilibrium parameters, pseudo-first and pseudo-second order kinetic models were also conducted^48^:

1b $$AE = AE_{e} \left[ {1 - \exp \left( { - kt} \right)} \right]\,\,\,\, \left( {\text{Lagergren Pseudo } - \text{ first order}} \right)$$

1c $$\frac{t}{AE} = \frac{1}{{V_{0} }} + \frac{1}{{AE_{e} }}t\,\,\,\,\, \left( {\text{Pseudo } - \text{ second order}} \right)$$

In these equations, $AE_{e}$ is the adsorption efficiency in equilibrium, $k$ is the pseudo-first order adsorption rate coefficient, and $V_{0}$ is related with the initial adsorption rate. Nonlinear fits of experimental data for models described by Eqs. (1b) and (1c) gave rise to $R^{2}$ values equal to 0.99933 and 0.99975, while $\chi^{2}$ values were 1.83 and 0.69, respectively. To test the validity of the models, F-test (significance $\alpha = 0.05)$^49^, Akaike’s information criterion (AIK) and Bayesian information criterion (BIC)^50^ were performed in software Origin 2019. The application of all the tests to Eqs. (1a)–(1c) clearly concludes that the hybrid model, Eq. (1a), is more likely to represent the correct kinetic behavior.

At pH 7 (optimum value to reach the maximum adsorption efficiency) the IB is mostly in its ionized form. However, at this pH, the surface of the nanoparticle will have mainly neutral character, so the procedure does not occur instantaneously, requiring 45 min to achieve maximum adsorption.

### Adsorption isotherms

**Figure 6:**
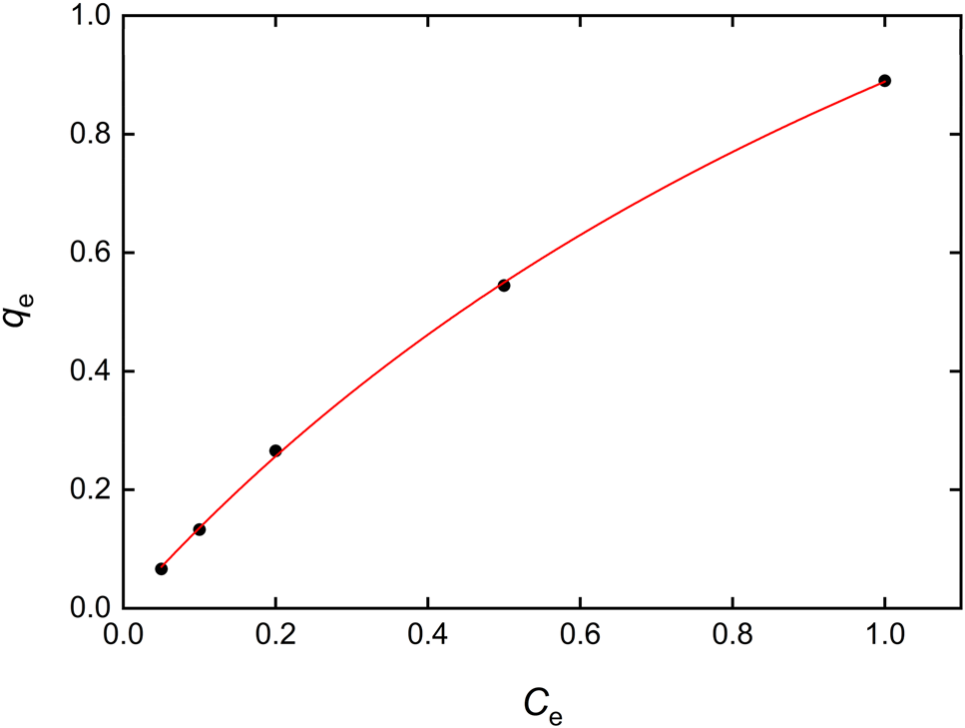
Langmuir isotherm plot $q_{e}$ vs. $C_{e}$ for IB adsorption to 7 mg Fe_3_O_4_@AgNPs at *T* = 298 K and pH = 7. Solid line represents the nonlinear fit to Eq. (2a).

The adsorption isotherm for the process was determined for several values of the equilibrium adsorption capacity $q_{e}$ (mg g^−1^) and the equilibrium concentration of adsorbate $C_{e}$ (mg L^−1^). A Langmuir isotherm model was employed, described by the following equation^51^:

2a $$\frac{1}{{q_{e} }} = \frac{1}{{q_{m} }} + \frac{1}{{K_{L} q_{m} C_{e} }}$$

where $q_{m}$ is the maximum adsorption capacity of adsorbent (mg g^−1^) and $K_{L}$ is the Langmuir adsorption constant (L mg^−1^). Figure 6 shows the dependence of $1/q_{e}$ vs. $1/C_{e}$ when 7 mg of Fe_3_O_4_@AgNPs (500 μL of suspension) are employed as adsorbent at pH = 7 and *T* = 298 K. The red solid line is the nonlinear fit to the Langmuir equation, $r^{2} = 0.99961$ and $\chi^{2} = 4.49 \times 10^{ - 5}$, proving that it is a suitable model for describing the equilibrium behavior of the adsorption experiment.

However, in order to deepen into the isotherm characterization, Freundlich and Temkin isotherms were also fitted to the experimental data:

2b $$\log (q_{e} ) = \log (K_{F} ) + \frac{1}{n}\log (C_{e} ) \left( {{\text{Freundlich}}} \right)$$

2c $$q_{e} = \frac{RT}{b}\ln \left( {K_{t} C_{e} } \right)\,\,\,\left( {{\text{Temkin}}} \right)$$

In these isotherm models, $K_{F}$ is Freundlich constant, $1/n$ is the adsorption intensity, $R$ is the gas constant, $T$ is the temperature and $K_{t}$ is Temkin isotherm constant. Nonlinear fits of data for models described by Eqs. (2b) and (2c) gave rise to $R^{2}$ values equal to 0.99622 and 0.92317, while $\chi^{2}$ were $4.34 \times 10^{ - 4}$ and 0.00998, respectively. For the comparison tests computed in Origin 2019, AIC and BIC clearly state that Eq. (2a), Langmuir, is more likely to characterize the equilibrium behavior for the adsorption process. F-test shows that it has not enough information to draw a conclusion. All the results may indicate that Langmuir isotherm characterizes the equilibrium for the adsorption experiment.

### Thermodynamic analysis

As mentioned in Sect. 2.3, Fe_3_O_4_@AgNPs is dissolved when the temperature is above 303 K. For temperatures equal to 298 and 303, the adsorption efficiency found gives rise to the same result, 93%. However, to complete the study, we performed a thermodynamic analysis of the adsorption process once the Fe_3_O_4_@AgNPs is dissolved. We studied temperatures *T* = 313, 323, 343, 353 and 363 K, for IB concentration $C_{e} = 0.002$ mg L^−1^ and Fe_3_O_4_@AgNPs mass equal to 0.007 g. Van’t Hoff equation enables us to determine the standard enthalpy, $\Delta H^{0}$ (J mol^−1^), and standard entropy, $\Delta S^{0}$ (J mol^−1^ K^−1^) corresponding to the adsorption process^51^:

3 $$ln\left( {K_{D} } \right) = \frac{{\Delta S^{0} }}{R} - \frac{{\Delta H^{0} }}{RT}$$

where $R$ is the gas constant (8.314 J mol^−1^ K^−1^) and *T* is the absolute temperature. Variable $K_{D}$ is the so-called distribution coefficient, defined as:

4 $$K_{D} = \frac{{q_{e} }}{{C_{e} }}$$

The standard Gibbs free energy $\Delta G^{0}$(kJ mol^−1^) is another thermodynamic variable of interest, since can give information about the nature of the adsorption process. Values of $\Delta G^{0}$ within the interval [− 20, 0] kJ mol^−1^ reveal a physisorption process, while [− 20, 0] kJ mol^−1^ are considered as characteristic for chemisorption^51^. $\Delta G^{0}$ can be determined from as follows:

5 $$\Delta G^{0} = - RTln\left( {K_{D} } \right)$$

**Figure 7:**
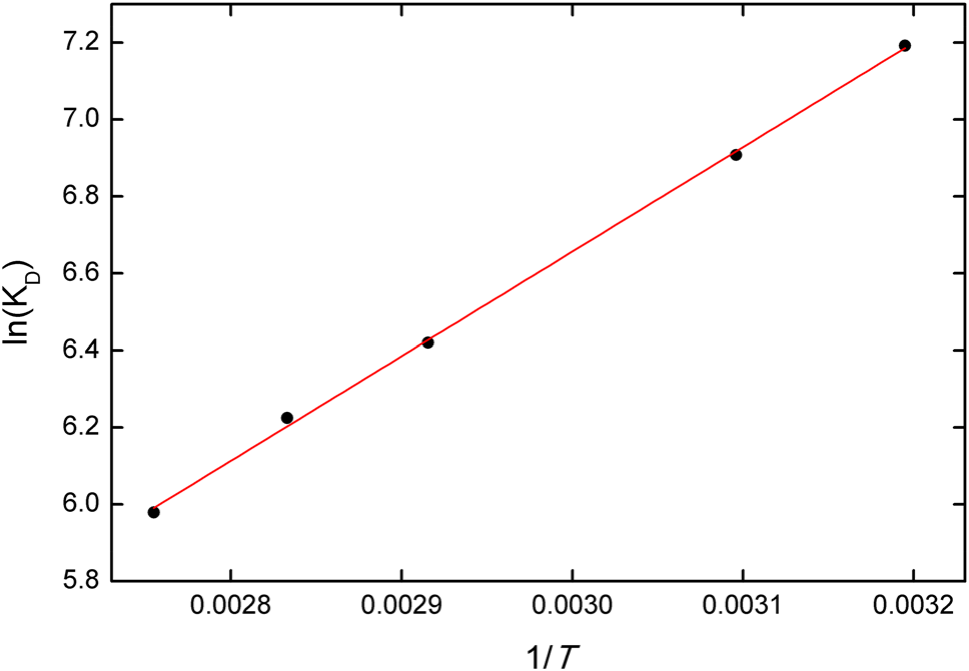
Van’t Hoff plot showing the dependence of $ln\left( {K_{D} } \right)$ on $1/T$ for IB adsorption to 7 mg of Fe_3_O_4_@AgNPs at *T* = 293 K, $C_{e} = 0.002$ mg L^−1^ and pH = 7. Solid line represents the linear fit to the experimental data.

Figure 7 shows the dependence of $ln\left( {K_{D} } \right)$ on $1/T$ for temperatures *T* = 313, 323, 343, 353 and 363 K. The red solid line represents the linear fit to the experimental data $r^{2} = 0.9989$. From Eq. (4) we determine , $\Delta H^{0} = - 22.57$ (kJ mol^−1^) and $\Delta S^{0} = 12.4$ (J mol^−1^ K^−1^), which reveals and exotermic process with an increase of randomness at solid/liquid interface for the adsorption procedure^51^. From Eq. (5) we determine that $\Delta G^{0}$ values lie within the range [− 18.71, − 18.03] kJ mol^−1^, indicative of a physisorption process.

### Ibuprofen adsorption in real water samples

The proposed method for the adsorption of ibuprofen in waters was applied to real water samples partially contaminated with this chemical compound. It was found that the concentration of the pollutant in these samples was low or not detected, so they were doped with a known concentration of ibuprofen (0.2 mg L^−1^) in order to check the viability of the proposed process. Adsorption efficiencies close to 93% were achieved in all cases, thus enabling the method as suitable for its application in real water samples. The results are summarized in Table 1.

**Table 1:** Results obtained after applying the method to real water samples.

Water sample	Ibuprofen concentration (µg L^−1^)^b^	Adsorption efficiency %	Adsorption efficiency after doping %
Wastewater^a^	2.31 ± 0.05	93.1	92.9
River water	0.62 ± 0.02	92.8	93.0
Drinking water	Not detected	–	93.1
Seawater	Not detected	–	92.8

^a^Water sample before being treated at the wastewater treatment plant in the city of Murcia, Spain.

^b^Each sample was analyzed by triplicate.

### Desorption and recycling studies

In order to carry out the desorption of the IB from the surface of the nanoparticles, 1 ml of a solution of nitric acid at pH 1 was used. For that value, interactions between adsorbent and adsorbate are weakened due to the effect of the ionic strength, as stated in “Effect of pH on IB adsorption”, thus favoring the desorption process. When the IB adsorption is carried out, after separation from the aqueous solution with the magnet, this solution is decanted. Then, 1 ml of the nitric acid solution is added and the mixture is sonicated for 3 min. After that, the adsorbent is removed with the magnet and the acid solution is quantified by high performance liquid chromatography, demonstrating that the IB total has been desorbed. Recycling studies were carried out showing that Fe_3_O_4_@AgNPs can be used during two additional successive adsorption cycles without losing adsorption capacity. In the fourth adsorption cycle, the capacity decreases down to 89.4%. The results are shown in Table 2.

**Table 2:** Recycling studies.

Adsorption cycle	Adsorption capacity (%)
1	93
2	93
3	93
4	89.4

Data for the four recycling cycles are shown.

## Conclusion

This work proposes a novel simple method for the adsorption of IB in water using Fe_3_O_4_@AgNPs. The results show a maximum IB removal efficiency of 93% from aqueous solutions, achieving the maximum adsorption at neutral pH and room temperature. The procedure takes 45 min and employs a dose of adsorbent equal to 7 mg in 500 µL of suspension, which can be completely removed from the medium using a magnet. The characterization of the adsorbent by means of microscopy, spectroscopy and calorimetry techniques reveal the presence of Ag in Fe_3_O_4_@AgNPs and the adsorption of IB. The adsorption equilibrium is characterized by a Langmuir isotherm. As a final remark, a simple method in acid media by using nitric acid pH = 1 has been proposed for the recycling and reuse of the adsorbent, which reaches 89.3% removal efficiency after three regenerations. The method presented in this work is suitable for removal of IB-like emergent pollutants from waters which cross the barriers of purification systems due to their low concentrations.

## Supplementary information

Supplementary Information
