# Supplementary material for: Magnetic core-modified silver nanoparticles for ibuprofen removal: an emerging pollutant in waters
Source: Sci Rep. 2020 Oct 26;10:18288. doi: 10.1038/s41598-020-75223-1 (PMC7588452; doi:10.1038/s41598-020-75223-1)
Supplement: Supplementary file 1 — Supplementary Information [file 41598_2020_75223_MOESM1_ESM.docx]

**Magnetic core-modified silver nanoparticles for ibuprofen removal. An emerging pollutant in waters**

*Yesica Vicente-Martínez; Manuel Caravaca; Antonio Soto-Meca;

Rubén Solana-González

University Centre of Defence at the Spanish Air Force Academy, MDE-UPCT, C/Coronel López Peña s/n, 30720 Santiago de la Ribera, Murcia, Spain.

E-mail address: [yesica.vicente@cud.upct.es](mailto:yesica.vicente@cud.upct.es)

**Supplementary Material**

**Fig. S1.** FTIR spectrum for Fe_3_O_4_@AgNPs after the adsorption process.


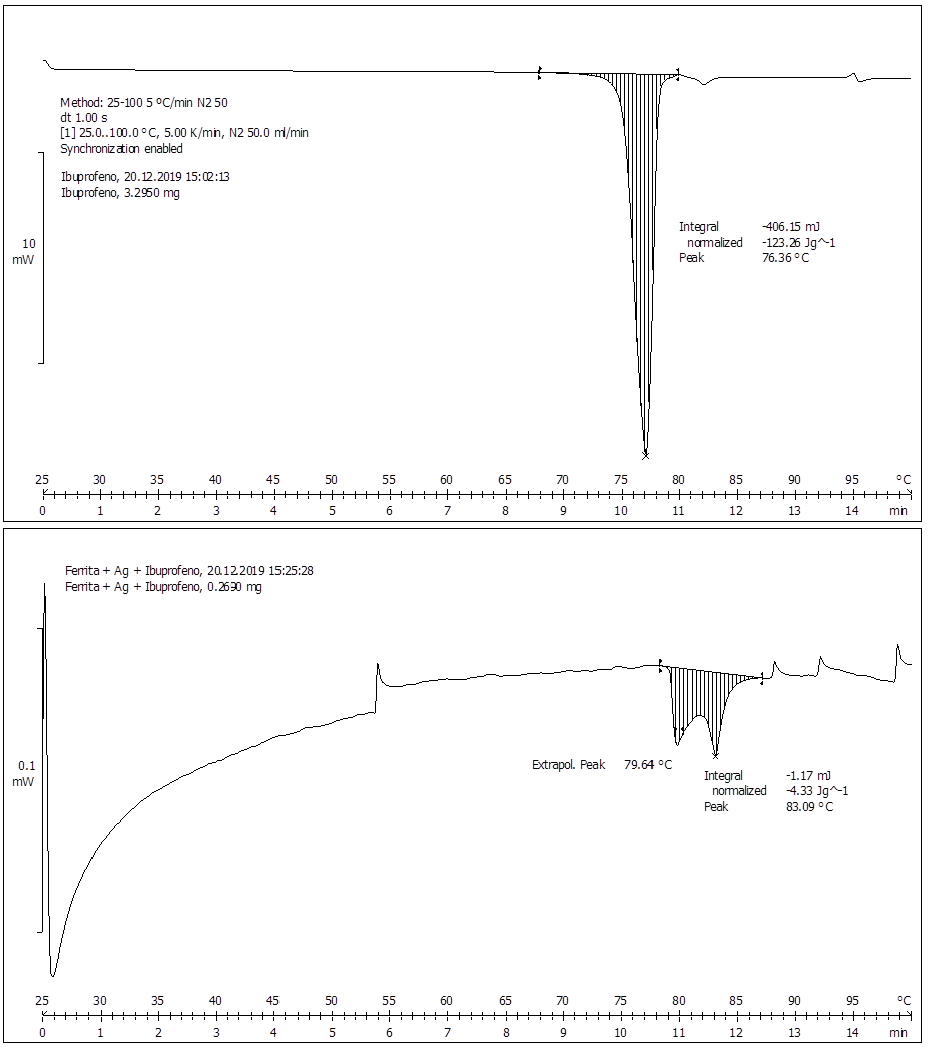


**Fig. S2.** DSC graphs of pure IB (top) and IB adsorbed onto Fe_3_O_4_@AgNPs (bottom).
